# Plate fixation strategies for fibular reconstruction following segmental mandibulectomy: A comparative study of double-barrel and single-barrel techniques

**DOI:** 10.1016/j.jpra.2026.03.004

**Published:** 2026-03-18

**Authors:** Kazuhiro Murakami, Nobuhiro Ueda, Yosuke Nakagawa, Miki Zaizen

**Affiliations:** a Department of Oral and Maxillofacial Surgery Nara Medical, University 840 Shijo-cho, Kashihara, Nara 634-8522, Japan; b Department of Oral and Maxillofacial Surgery, Hattiori Memorial Hospital, Nara, Japan; c Department of Oral and Maxillofacial Surgery, Division of Oral Pathogenesis and Disease Control, Asahi University Hospital, Gifu, Japan

**Keywords:** Mandibular reconstruction, Double-barrel fibula flap, Finite element analysis, Custom three-dimensional plate, Mechanical stability

## Abstract

**Purpose:**

Mandibular defects following oncological resection present significant aesthetic and functional challenges due to the loss of bone and soft tissue. Immediate reconstruction is crucial to prevent complications such as malocclusion, mandibular deviation, temporomandibular joint dysfunction, and impaired mastication, swallowing, and speech. Although the fibula flap is widely used, its limited vertical height complicates alveolar arch restoration in dentate mandibles. The double-barrel fibula flap addresses this limitation; however, the biomechanical stability of various fixation methods remains underexplored.

**Methods:**

Finite element analysis was performed using computed tomography data from a patient with T4 mandibular gingival carcinoma. Five fixation configurations were evaluated: four short plates, one long plate with two short plates, two box plates, a custom-designed long box plate, and a single-barrel model. Simulated masticatory forces and boundary conditions were applied, with material properties assigned through Hounsfield unit conversion and established literature values.

**Results:**

Stress concentrated at the fibula–mandible junction, particularly at the proximal interface and along the inferior border. The long + two-short-plate configuration demonstrated the lowest stress. Custom plates reduced displacement and altered stress distribution. Single-barrel models consistently exhibited higher stress and displacement.

**Conclusions:**

Integrating the fibula with the proximal and distal mandibular segments using a long plate positioned along the inferior border enhances stability. Incorporating a three-dimensional plate structure may further reduce displacement. Double-barrel reconstruction offers superior biomechanical stability compared with single-barrel techniques.

## Introduction

Mandibular defects resulting from oncological treatment pose significant aesthetic and functional challenges because they involve the loss of both bone and soft tissue. Immediate reconstruction is essential to preventing complications such as malocclusion, mandibular deviation, temporomandibular joint (TMJ) dysfunction, and soft tissue retraction.^1^ These complications can lead to functional impairments, including difficulties in mastication, swallowing, and speech.^2^ Reconstruction of mandibular continuity defects following tumor resection using free vascularized bone flaps is a well-established treatment approach. Various techniques have been developed, and multiple donor sites, including the iliac crest, fibula, scapula, and radius, have been reported to yield favorable outcomes.^3^, ^4^, ^5^, ^6^, ^7^

The fibula flap, first introduced by Hidalgo in 1989 for mandibular continuity reconstruction, offers several advantages, including adequate bone length, reliable vascularization, a long vascular pedicle, and suitable dimensions for implant placement.^3^^,^^8^, ^9^, ^10^ However, its limited vertical height presents challenges in restoring the alveolar arch, particularly in dentate mandibles.^11^ To address this limitation, the double-barrel fibula flap technique was developed.^12^

Double-barrel fibula reconstruction enables simultaneous restoration of mandibular height and contour, offering both aesthetic and prosthetic advantages through a relatively straightforward technique.^13^ Favorable outcomes have also been reported for postoperative implant placement in the reconstructed regions.^2^ Nevertheless, few studies have investigated the mechanical aspects of different fixation methods and their stability in fibular reconstructions, and the lack of comparative data has hindered the development of optimized fixation strategies for mandibular reconstruction.

The double-barrel technique is hypothesized to provide superior stability due to a larger bone contact surface compared with the single-barrel approach. Furthermore, fixation using a custom-designed three-dimensional (3D) plate may yield better mechanical outcomes than conventional plates. This study evaluates the effectiveness of various fixation methods, including a tailor-made 3D plate, for double-barrel reconstruction and compares the mechanical stability of single-barrel and double-barrel models.

## Materials and methods

This study was conducted in accordance with the principles of the Declaration of Helsinki and was approved by the Ethics Committee of Nara Medical University (Approval No 3930). All patients were fully informed of the study’s purpose and provided written informed consent before inclusion. Additionally, the study adhered to the Strengthening the Reporting of Observational Studies in Epidemiology (STROBE) guidelines.

### Patient

The finite element (FE) model used in this analysis was based on computed tomography (CT) data from a male patient in his 50 s diagnosed with T4 right mandibular gingival carcinoma. The patient was scheduled to undergo segmental mandibulectomy followed by double-barrel fibula flap reconstruction.

### Finite element analysis

#### Computed tomography imaging for surgical planning

CT imaging was performed 1 month before surgery. Mandibular CT scans were acquired using 1-mm-thick slices spanning from the submental region to the supraorbital rim. Similarly, fibular CT scans were obtained with 1-mm-thick slices extending from the proximal to the distal fibular stump. To minimize radiation exposure, the scanning range was restricted to the minimum necessary area, exposure time was reduced, and radiation shielding equipment was provided during imaging. All CT images were converted to Digital Imaging and Communications in Medicine (DICOM) format.

#### Finite element model construction

**Figure 1:**
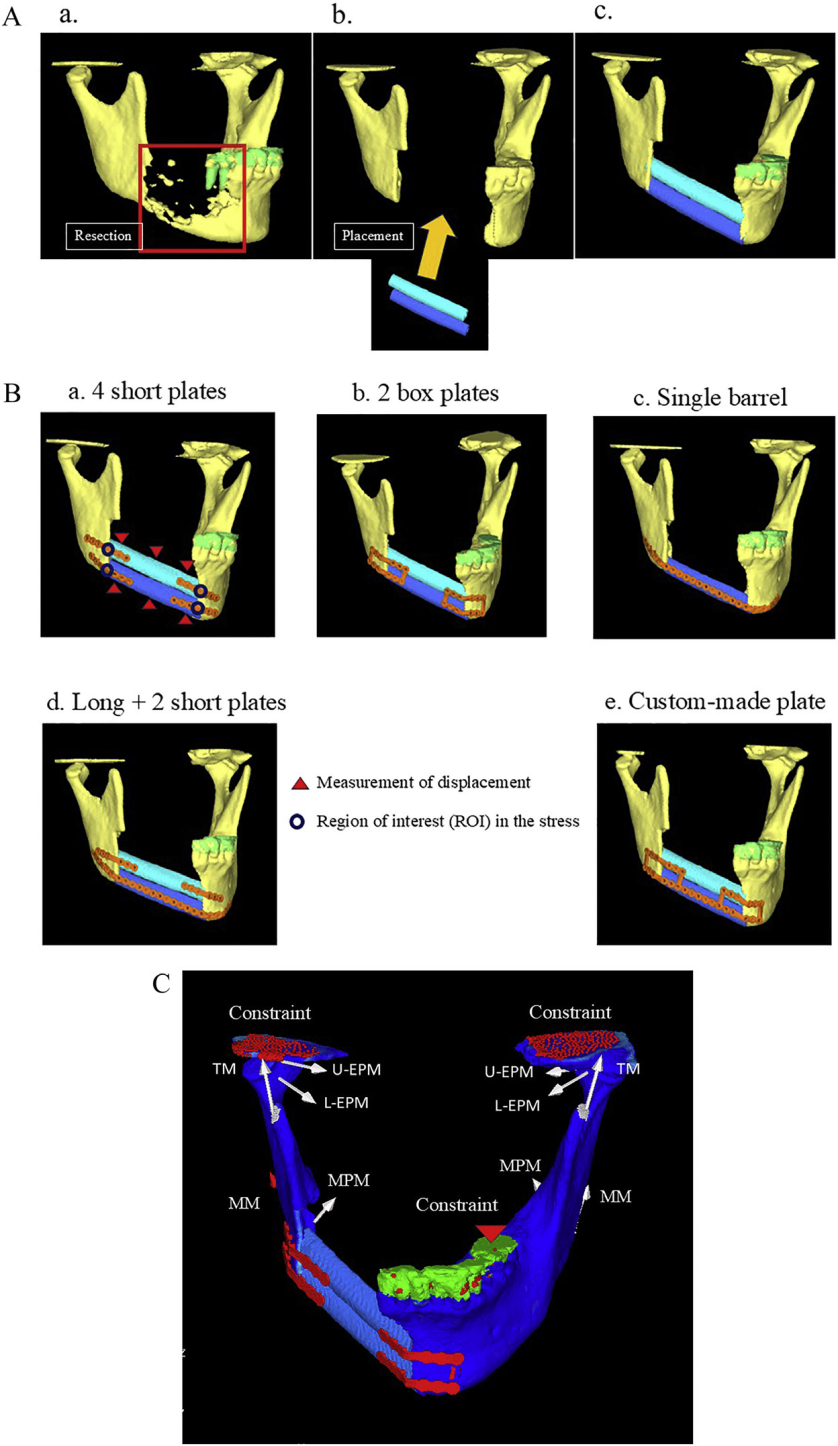
A: Construction of the finite element (FE) model. a. Conversion of the patient’s computed tomography (CT) images. b. Virtual segmental resection on the FE model. c. Reconstruction of the mandibular defect using the fibula in the FE model. B: Plate fixation between the mandible and fibula. C: Muscle forces and boundary conditions applied to the FE Model. U, Upper; L, Lower; MM, Masseter Muscle; TM, Temporal Muscle; MPM, Medial Pterygoid Muscle; EPM, External Pterygoid Muscle.

The DICOM data were used to construct the FE model (Figure 1Aa). The regions of interest (ROIs) included the entire mandible and a portion of the temporal bone encompassing the glenoid fossa. Virtual segmental resection of the mandible was performed on the model (Figure 1Ab), and the fibula was digitally trimmed on the 3D model to fit the defect (Figure 1Ac). Finally, both single-barrel and double-barrel fibular grafts were positioned within the mandibular defect for subsequent analysis.

#### Creation of a double-barrel fibula reconstruction model

The patient’s fibula was virtually segmented using computer-aided design (CAD) software to match the mandibular defect and positioned at the site of segmental resection. A 0.5-mm bone gap was intentionally created between the fibula and mandible to simulate clinical conditions.

#### Four types of plate fixation were evaluated to secure the mandible and fibula

**1. Conventional plate fixation**

Conventional fixation plates were designed using the Trauma One™ Lorenz® Plating System (Zimmer Biomet, Warsaw, IN, USA). The plates included a 2.0-mm-thick 6-hole plate with a gap and a 24-hole straight plate. Three-dimensional reconstruction of the mandibular plate was performed using Metasequoia (Tetraface Inc., Japan), a polygon-based CAD modeling software. Two fixation configurations were tested:

• A double-barrel model fixed with four short plates (Figure 1Ba).
• A double-barrel model with one 24-hole straight plate (long plate) along the inferior mandibular border and two short plates securing the proximal and distal ends of the upper fibular segment (Figure 1Bd).

**2. Patient-specific custom-made plate fixation**

**Two custom-designed plates were independently developed:**

• Box-shaped plate: Constructed by adding vertical bars to the terminal holes of two 6-hole gap plates, forming a box-shaped configuration (Figure 1Bb).
• Original custom-made plate: Created by integrating box-shaped structures at both ends of a 24-hole straight long plate using two 6-hole gap plates and one 24-hole straight plate (Figure 1Be).

All plates were fixed using virtual 3D screws modeled in CAD software, based on 2.0 × 14 mm X-Drive Locking screws (Zimmer Biomet, Warsaw, IN, USA). For the 6-hole and box plates, bicortical screws were placed in all available holes. For the 24-hole long plate, screws were inserted bicortically into three anterior and three posterior holes adjacent to the fibula–mandible junction. In the original long plate with box-shaped ends, screws were placed in all holes within the box-shaped regions.

#### Creation of a single-barrel fibula reconstruction model

A single fibular segment was positioned along the inferior mandibular border and secured using a 24-hole straight long plate (Figure 1Bc).

### Loading and boundary conditions

All models were subjected to identical loading and boundary conditions. The loading condition simulated unilateral molar clenching on the non-resected side, incorporating forces generated by the masticatory muscles, including the masseter, temporalis, lateral pterygoid, and medial pterygoid muscles (Table 1). The relative magnitudes of these muscle forces were derived from previous studies.^14^ For boundary conditions, the lateral surface of the temporal bone and the occlusal surface at the center of the crown of the left first molar were fully constrained in all directions (Figure 1C).

**Table 1:** Loading forces and directions.

	Force		Direction					
	Right	Left	Right			Left		
	N		cos-x	cos-y	cos-z	cos-x	cos-y	cos-z
Masseter	163.2	195.8	−0.212	−0.367	0.906	0.212	−0.367	0.906
Medial pterygoid	79.7	111.6	0.486	−0.373	0.791	−0.486	−0.373	0.791
Temporalis	185.2	223	−0.222	0.500	0.837	0.222	0.500	0.837
External pterygoid (upper)	43.5	20.1	0.761	−0.645	0.074	−0.761	−0.645	0.074
External pterygoid (lower)	18.7	8.6	0.630	−0.757	−0.174	−0.630	−0.757	−0.174

## Calculations

To assess bone heterogeneity, the mechanical properties of each FE were derived from Hounsfield unit values. Young’s modulus was calculated using the equations proposed by Keyak et al.,^15^ while Poisson’s ratio was uniformly set to 0.4 across all bone elements. The Poisson’s ratio and Young’s modulus were set to 0.19 and 105.9 GPa, respectively, for the titanium plate, and 0.28 and 108.9 GPa for the titanium screw. For dental tissues, the values were 0.3 and 50 GPa for enamel, and 0.3 and 10.7 GPa for dentin.^16^

### Generation of von Mises stress in the plate

The ROI for each plate was defined as a 5-mm zone located directly above the fibula–mandible junction, where stress concentration was maximal. Within this ROI, the average von Mises stress was calculated for all elements within a 1-mm radius centered on the element exhibiting the maximum stress (Figure 1B).

### Fibular displacement

Fibular displacement was evaluated at six points: three on the lower surface (center of the underside and 10 mm from the proximal and distal ends) and three corresponding points on the upper surface (Figure 1B).

Von Mises stress, tensile stress, compressive stress, and displacement values described above were compared across reconstruction types.

## Results

### Stress distribution

Von Mises stress in the plates was predominantly concentrated above the fibula–mandible junction, with higher stress observed in the proximal plate than the distal plate. Comparisons between upper and lower plates revealed that the lower plate, positioned along the inferior mandibular border, showed greater stress.

**Figure 2:**
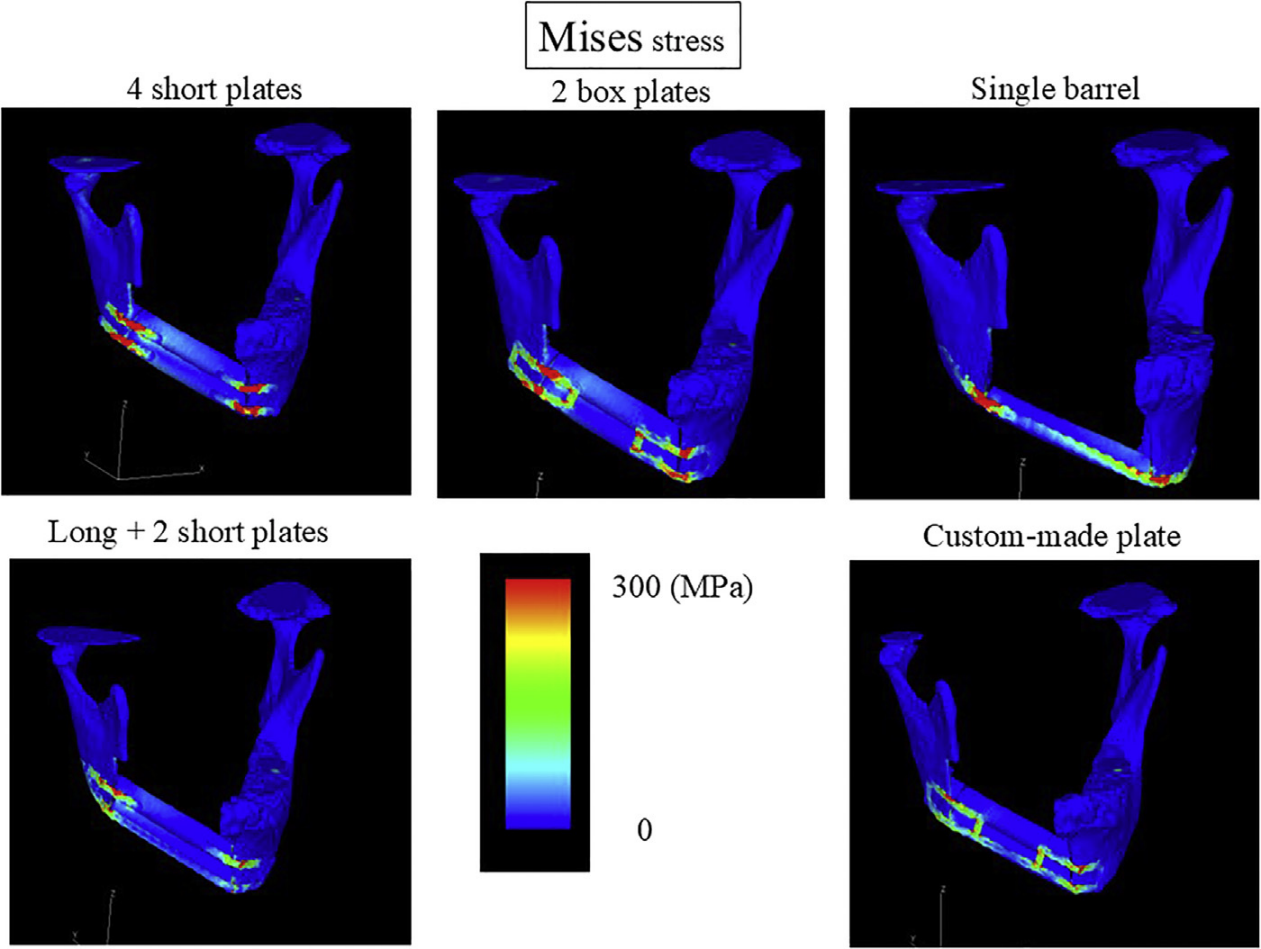
Distribution of von Mises stress across five model types.

Connecting the upper and lower plates in a box-shaped configuration mitigated stress in the four-plate fixation model. In the two designs incorporating this box structure (BOX and the original custom plate), stress concentration was observed along the vertical bars of the BOX (Figure 2).

**Figure 3:**
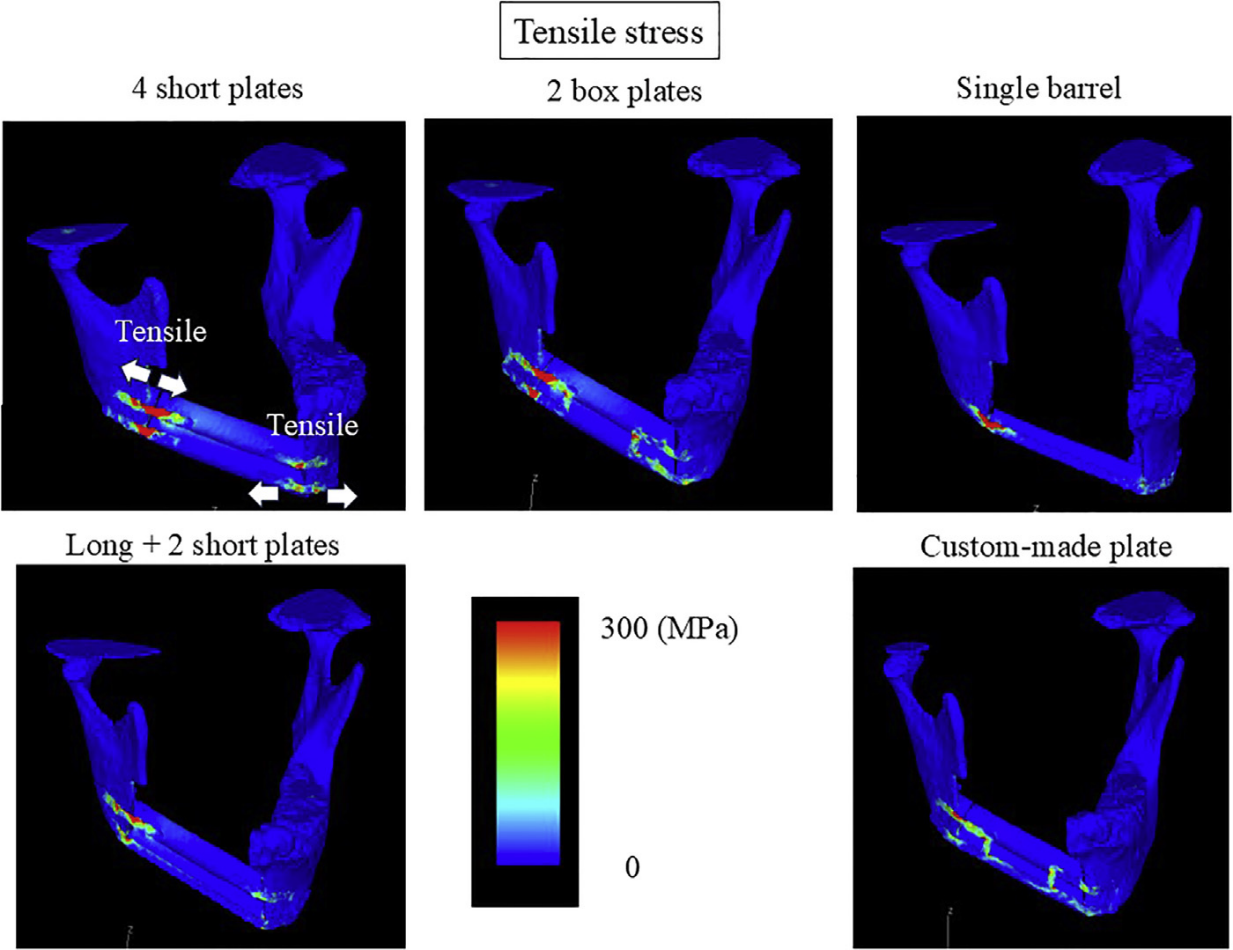
Distribution of tensile stress across five model types.

**Figure 4:**
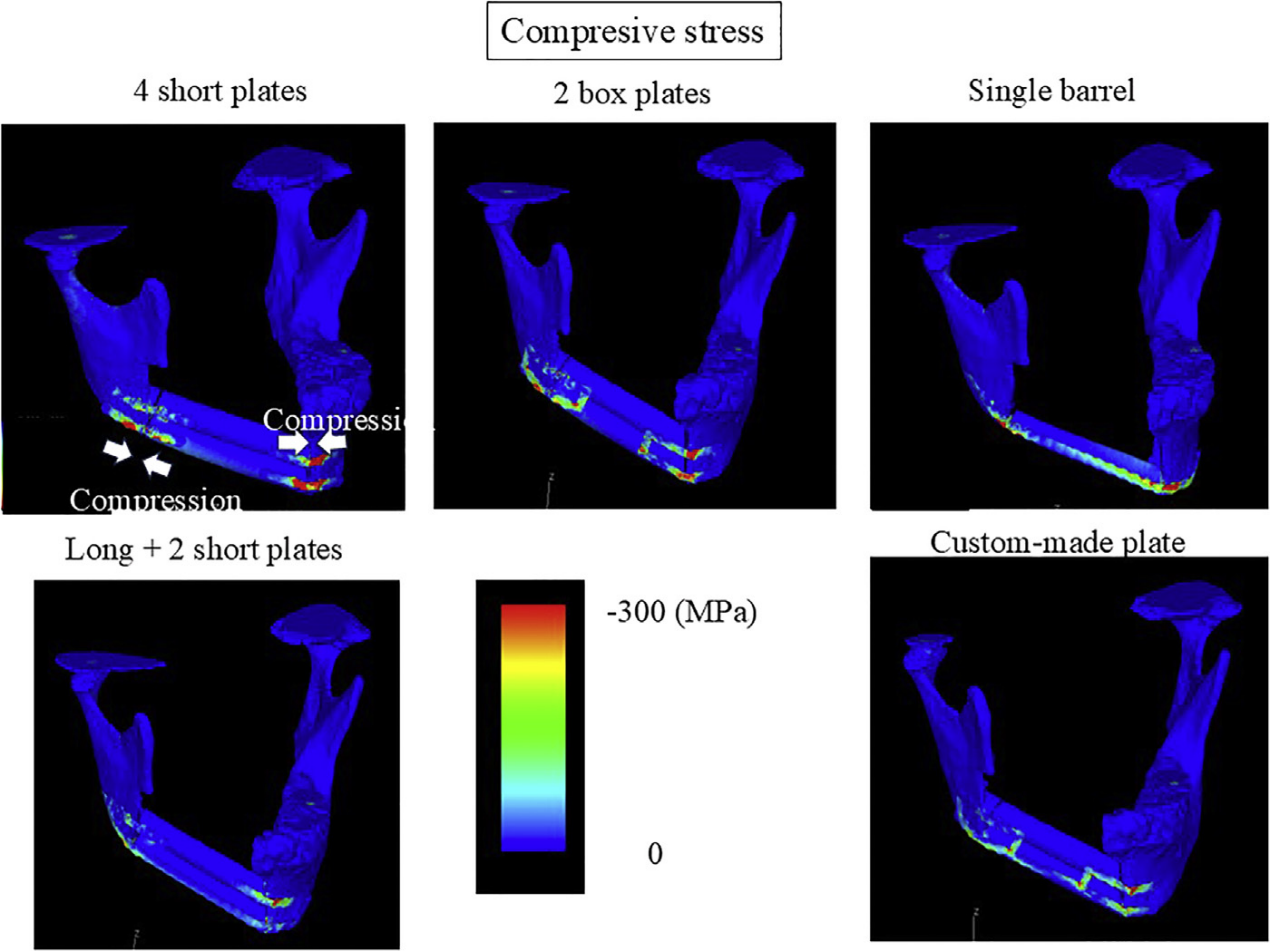
Distribution of compressive stress across five model types.

Tensile stress increased in the upper plate connecting the proximal mandibular fragment to the fibula and in the lower plate connecting the distal mandibular fragment to the fibula following segmental resection (Figures 3 and 6). Conversely, compressive stress was concentrated in the lower plate connecting the proximal mandibular fragment to the fibula and in the upper plate connecting the distal mandibular fragment to the fibula (Figures 4 and 6).

However, the introduction of a complex 3D structure incorporating screws, as in the BOX or original plate, altered the distribution and interaction between tensile and compressive stress lines (Figure 3, Figure 4).

### Comparison of maximum von Mises stress in the region of interest for each plate type

In the double-barrel fixation models, the configuration with four short plates exhibited the highest stress across all plate types, whereas the combination of one long plate and two short plates produced the lowest stress. Moreover, stress consistently appeared to be more pronounced in the proximal region than in the distal region throughout the analysis.

Comparison of stress distribution between the upper and lower plates showed that placing a plate along the inferior mandibular border helped equalize stress across each region of interest and reduce overall stress concentration. However, the custom-made plate generated slightly higher stress than the configuration using one long plate with two short plates.

**Figure 5:**
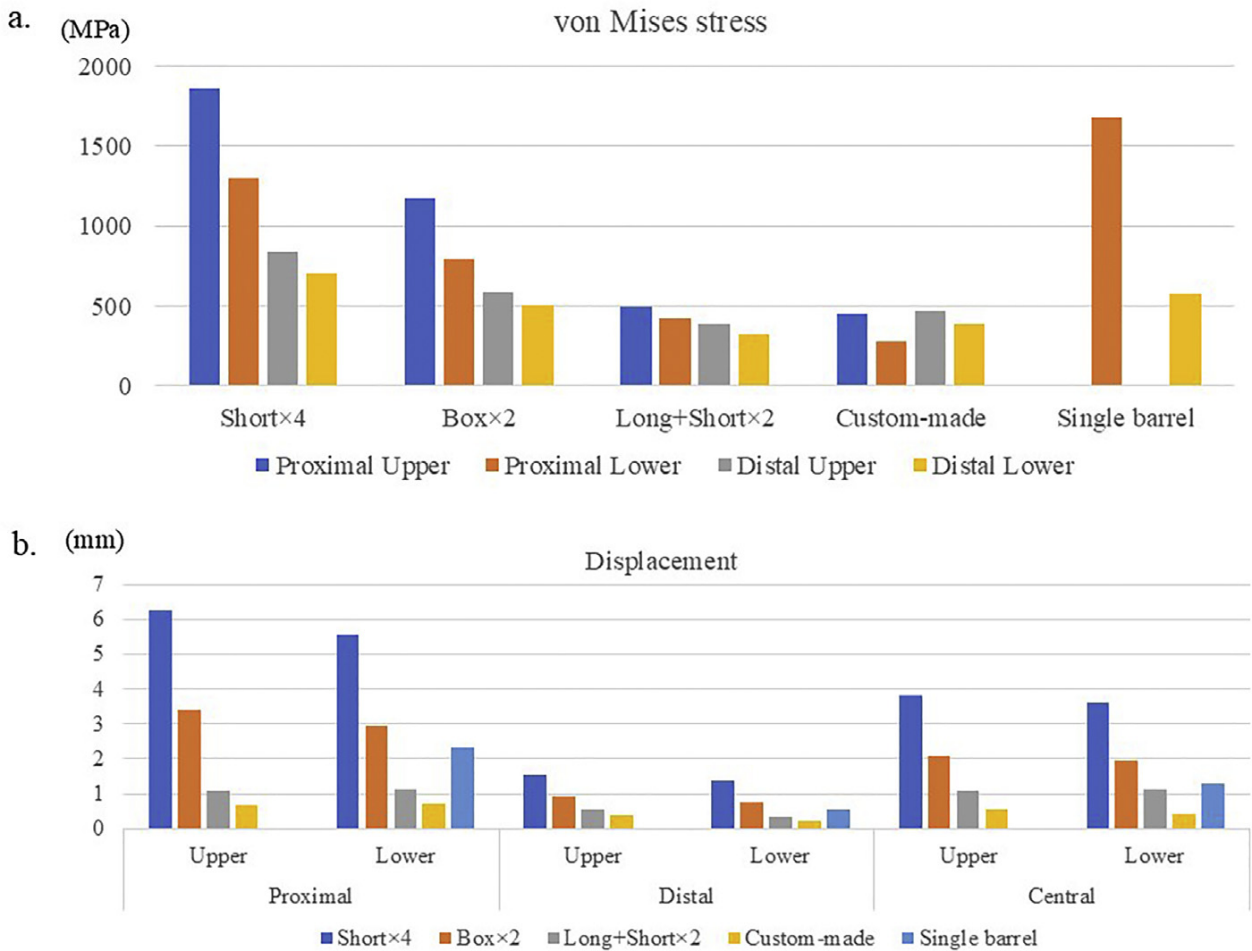
Comparative analysis of stress and displacement. a. Maximum von Mises stress across plate types and fixation methods. Double-barrel fixation using one long and two short plates produced the lowest stress, whereas the four-short-plate configuration generated the highest stress. Plating along the lower mandibular border reduced and balanced stress distribution. Single-barrel designs consistently exhibited higher stress levels despite integration. b. Fibular displacement across reconstruction designs. Proximal fibular displacement was generally greater than distal displacement. In the four-short-plate design, the lower fibula showed more proximal displacement than the upper fibula, whereas other designs showed no regional differences. Among double-barrel models, displacement followed the order: proximal lower > proximal upper > distal upper > distal lower, with the upper fibula showing greater distal displacement. Overall displacement ranked as follows: four short plates > single-barrel > box plates > long + short plates > custom-made plates.

In the single-barrel fixation models, the plate generated greater stress than in the double-barrel configurations. Even with a long plate positioned along the inferior mandibular border to connect the three segments, the resulting stress was nearly equivalent to the highest values observed among all configurations (Figure 5a).

### Comparison of maximum tensile and compressive stress in the region of interest for each plate type

**Figure 6:**
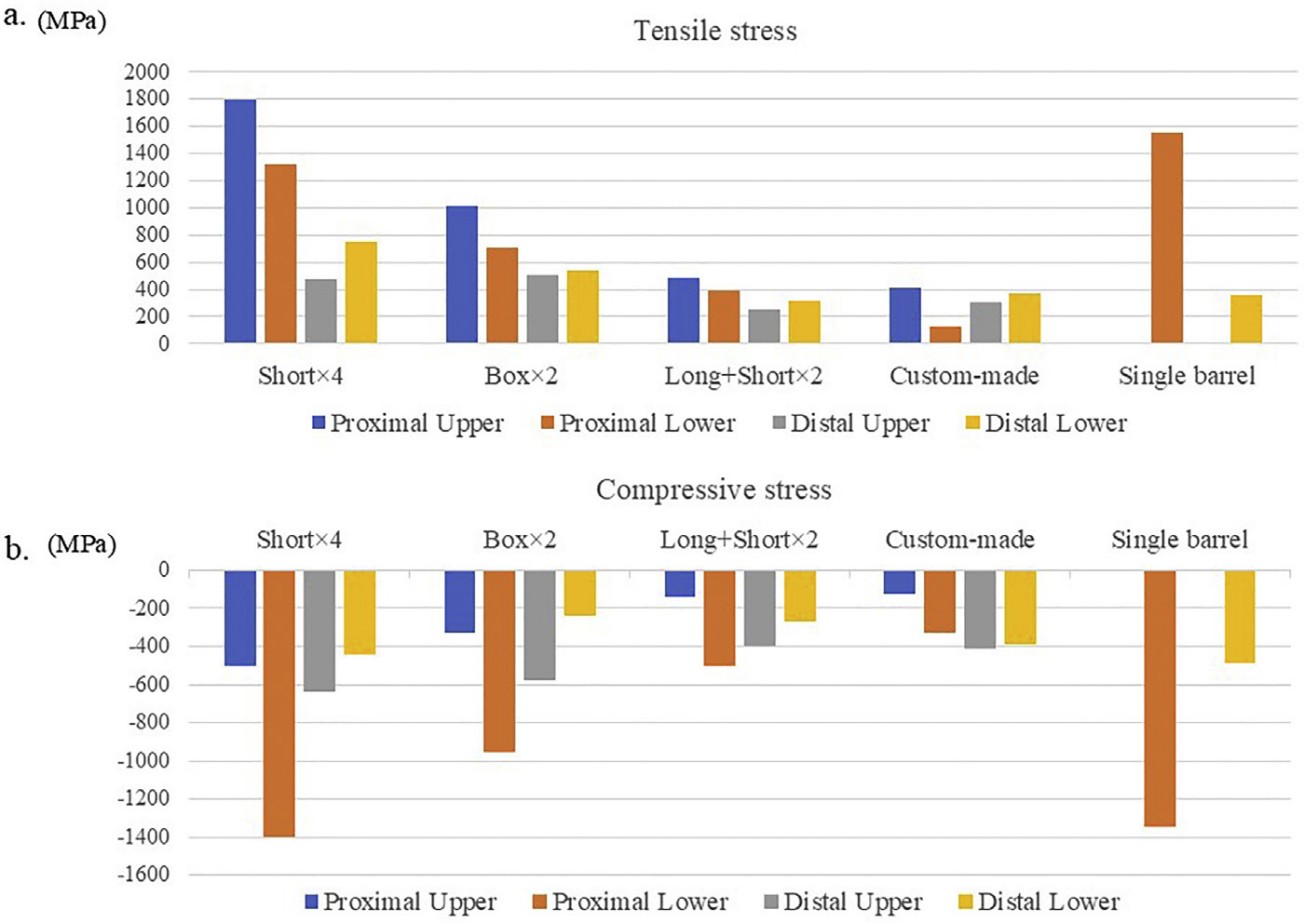
Comparison of tensile and compressive stress across models. Stress values typically followed the order: proximal lower > distal upper. The relative ranking of proximal upper and distal lower regions changed when the lower mandibular border was unified with a long plate. Custom-made plates exhibited a distinct stress distribution pattern consistent with tensile stress behavior.

For tensile stress, most plate configurations exhibited the following pattern: proximal upper > proximal lower > distal lower > distal upper. However, this pattern was altered when custom-made plates were used (Figure 6a).

Under compressive stress, conventional plates generally exhibited the following pattern: the proximal lower region showed the highest stress, while the proximal upper region showed the lowest stress. However, the ranking between the proximal upper and distal lower regions changed when using the two types of custom-made plates (box-type and original custom-made designs). As with tensile stress, custom-made plates exhibited a distinctly different stress distribution compared with conventional designs (Figure 6b).

### Comparison of fibular displacement

Fibular displacement was greater in the proximal region than in the distal region. In all configurations, the upper surface of the fibula exhibited greater displacement than the lower surface. However, except for the four-short-plate configuration, other plate designs showed no remarkable difference between upper and lower displacements in the proximal region.

Across all double-barrel configurations, displacement followed the pattern: proximal lower > proximal upper > distal upper > distal lower. For distal displacement, all four double-barrel types exhibited greater displacement in the upper region than in the lower region.

**Figure 7:**
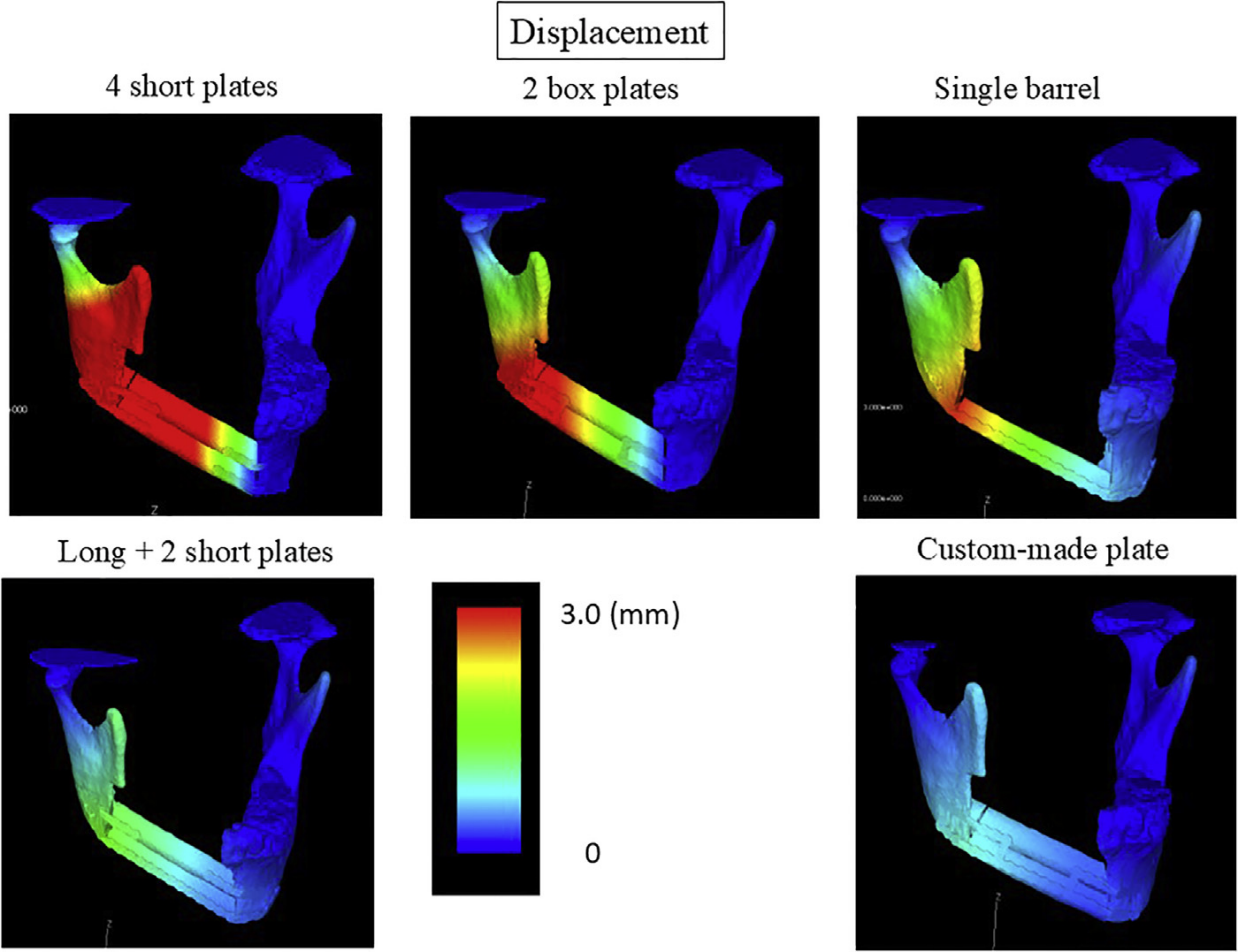
Distribution of compressive stress-related displacement across five model types.

Regarding displacement by plate type, the magnitude decreased in the following order: four short plates > single-barrel > two box plates > one long plate with two short plates > custom-made plates. Among all configurations, the single-barrel model exhibited the second-highest displacement (Figure 5, Figure 7).

## Discussion

In this study, we selected a case in which the mandibular height at the segmental resection stump was approximately equal to the height of the fibular reconstruction using the double-barrel technique. In atrophic mandibles, double-barrel reconstruction can produce a vertical mismatch that may affect the restoration of the mandibular arch, thereby compromising facial aesthetics.^13^^,^^17^

Since the focus of this study was on fibular fixation methods, cases with comparable mandibular and fibular heights were specifically selected.

### Stress

When the three segments along the inferior mandibular border in a double-barrel reconstruction are not properly integrated, high stress is generated in each plate. However, integrating these segments distributes the stress concentration more evenly and reduces stress within the ROI across all plate types. This is because the inferior mandibular border is a critical structure for mandibular strength and represents the most important area for reconstruction.^18^^,^^19^ Accordingly, fixation using four short plates or two box plates resulted in intersegmental flails, even when bicortical screws were applied.

In this study, a 0.5-mm bone gap was defined between the fibula and mandible. This strict setting reflects the clinical difficulty of achieving close contact between the resected surfaces of the donor fibula and the recipient mandible. Therefore, in actual clinical situations, if partial contact and soft tissue support are present, the stress on the plate and displacement of the fibula may be lower than those observed in the FE model analysis.

In mandibular fracture reconstruction, the inferior mandibular border typically serves as the compression zone, while the region near the alveolar bone functions as the tensile zone, according to Champy’s ideal osteosynthesis line.^20^ When a double-barrel reconstruction is performed following segmental resection, mandibular continuity is disrupted. Consequently, compressive stress is applied to the lower proximal and upper distal plates, whereas tensile stress is applied to the upper proximal and lower distal plates, thereby forming diagonal stress lines. Therefore, in double-barrel reconstruction, both the upper and lower fibular segments must be fixed to the resected mandibular surfaces at four points, proximally and distally. In cases where an iliac or other donor bone is used instead of the fibula, fixation along both the upper and lower lines is considered necessary.

However, when complex 3D structures, such as box-shaped or custom-made plates with bicortical screws, are used, the relationship between tensile and compressive stress lines is altered. In two-dimensional structures, stress can be considered primarily in the axial direction. In contrast, 3D configurations increase the stress tensor complexity, and even a unidirectional load can induce diagonal stress and internal deformation. This phenomenon may disrupt the theoretical linear stress–strain relationship.

Furthermore, larger plate volumes can increase the risk of postoperative mucosal or skin exposure.^21^^,^^22^ To minimize this risk, a box-shaped structure was designed by joining the ends of the upper and lower plates. FE analysis of the box-shaped and custom-made plates revealed stress concentration along the vertical bars of the box. Although this structure was less effective than a single long plate in integrating the three segments along the inferior mandibular border, the vertical bars helped integrate the upper and lower fibulae, which may have slightly improved stability. These findings suggest that integrating the three segments with a single long plate is essential for stabilizing the reconstructed area.

### Displacement

FE analysis revealed a minimum displacement of 0.6 mm in the central upper region of the fibula when using the original custom-made plate. However, this value is likely to be lower in clinical practice due to contact between the mandible and fibula, as well as support from surrounding soft tissue. Therefore, the reconstructed area is expected to remain stable provided no significant external load is applied.

In this study, the production of box-shaped and custom-made plates would incur additional manufacturing costs in actual clinical settings. Although the virtually designed custom-made plate would be ideal, the long-short plate fixation appears to be a more cost-effective option.

### Limitations

The case used in this study involved a typical unilateral molar segmental resection. However, stress and displacement patterns may vary significantly depending on the location and extent of the segmental defect. Since such cases have frequently been the focus of previous investigations,^2^^,^^5^^,^^17^, ^18^, ^19^ they have strong potential to serve as representative models for clinical reconstruction planning. Nevertheless, because tumor location and extent differ among patients, it will be necessary to develop a system capable of rapidly analyzing the optimal reconstruction method for each individual case.

Additionally, the occlusal setting in this study was limited to clenching the molars on the contralateral side of the resection, and the applied muscle forces and directions were based on representative values reported by Korioth.^14^ Therefore, patient-specific data will be essential for constructing individualized simulation systems.

Furthermore, the TMJ structure was simplified in this study. Although this simplification is unlikely to have significantly affected the overall trends in displacement and stress distribution in the FE analysis, a more anatomically accurate TMJ model may be required for highly precise simulations.

## Conclusion

In fibula reconstruction following segmental mandibular resection, integrating the fibula with the proximal and distal mandibular segments using a single long plate positioned along the inferior mandibular border is recommended for enhanced stability. Incorporating a 3D structure into the plate may further reduce fibular displacement. Moreover, double-barrel reconstruction provides substantially greater mechanical stability of the reconstructed area than single-barrel reconstruction.

## Availability of data and materials

The datasets generated and/or analyzed during this study are not publicly available due to the risk of data misuse or unauthorized use before publication. However, they are available from the corresponding author upon reasonable request after publication.

## Funding

This study received no funding.

## Ethics approval and consent to participate

This study was conducted in accordance with the Declaration of Helsinki and was approved by the Ethics Committee of Nara Medical University (Approval No. 3930). Finite element analysis was performed using anonymized computed tomography data from a single patient diagnosed with T4 mandibular gingival cancer. The patient was fully informed of the study objectives and provided written informed consent before participation. As the dataset was derived from a single individual and was fully anonymized, the risk of identification is minimal. This study also adhered to the Strengthening the Reporting of Observational Studies in Epidemiology (STROBE) guidelines.

## Consent for publication

Not applicable.

## Author contributions

Kazuhiro Murakami contributed to the conception, design, data acquisition, and interpretation; performed the statistical analysis; and drafted and critically revised the manuscript. Nobuhiro Ueda contributed to the conception and design of the manuscript and critically revised it. Yosuke Nakagawa contributed to the drafting and critical revision of the manuscript. Miki Zaizen contributed to drafting and critical revision of the manuscript. All authors gave their final approval and agreed to be accountable for all aspects of this work.

## Declaration of competing interest

The authors declare no competing interests concerning the authorship and/or publication of this article.
